# FunGen‐xQTL atlas: a multi‐omic atlas of quantitative trait loci in human brain and cerebrospinal fluid for target identification and prioritization in Alzheimer’s disease

**DOI:** 10.1002/alz.092045

**Published:** 2025-01-03

**Authors:** Yuk Yee Leung

**Affiliations:** ^1^ Department of Pathology and Laboratory Medicine, University of Pennsylvania Perelman School of Medicine, Philadelphia, PA USA

## Abstract

**Background:**

To gain a deeper understanding of underlying molecular mechanisms in genomic regions associated with Alzheimer’s disease (AD), the National Institute on Aging (NIA) launched the Alzheimer’s Disease Sequencing Project (ADSP) Functional Genomics Consortium (FunGen‐AD) in 2021.

**Method:**

The first effort of this collaboration, coordinated by the NIA Genetics of Alzheimer’s Disease Data Storage Site (NIAGADS), aggregated functional genomics (FG) data from 5 cohorts, including ∼3,000 samples of European (EA) and African ancestries (AA). We used this data to map Quantitative Trait Loci (xQTL) on AD‐specific human tissues and cells, providing insights into how non‐coding genetic variants contribute to AD risk. We developed reproducible computational pipelines to uniformly process the data, generating xQTL from >30 datasets profiled from human brain, cerebrospinal fluid, myeloid cells, and whole blood. The genetic data was processed exactly as the ADSP WGS datasets (GRCh38, same variant/gene annotations). For each QTL dataset, performed quality control and imputed missing data, inferred hidden confounders, and conducted genome‐wide association testing for cis‐ and trans‐acting QTLs. We also performed statistical fine‐mapping to identify potential causal variants. The consolidated summary statistics contain effect alleles normalized to the GRCh38.

**Result:**

Overall, this resource yielded >10 million cis‐ and trans‐QTL on DNA methylation, histone acetylation, RNA expression, RNA splicing, proteins, and metabolites. To support the identification of potential causal variants for molecular traits and AD, we have created a multi‐ancestry linkage disequilibrium reference panel using ADSP WGS data from >16,000 individuals of self‐reported EA and >5,000 individuals of self‐reported AA.

Beyond this unique FunGen‐xQTL atlas, other FG datasets generated by other non‐ADSP members are needed for AD genetics analyses. We developed the Harmonization and Integration Pipeline for Functional Genomics (hipFG) to harmonize all FunGen‐xQTL data together with other public resources such as GTEx, eQTL catalog, and metaBrain. Using controlled vocabularies, hipFG produces a standardized metadata describing all outputs. This pipeline is used to expand FILER (FunctIonaL gEnomics Repository), a framework for querying large‐scale genomics knowledge with a scalable genomic search and querying interface.

**Conclusion:**

The FunGen‐xQTL resource will be made publicly available through Sage Bionetworks and NIAGADS.

